# A Curious Case of a Floating Gallbladder

**DOI:** 10.7759/cureus.88207

**Published:** 2025-07-17

**Authors:** Ankita Swarnkar, Sundeep Selvamuthukumaran, B. V. Sreedevi, Pola Govardhan Kumar, Mahesh K.G

**Affiliations:** 1 General Surgery, Sree Balaji Medical College and Hospital, Chennai, IND

**Keywords:** floating gallbladder, gallbladder anomaly, gallbladder torsion, laparoscopic cholecystectomy, wandering gallbladder

## Abstract

A floating gallbladder is a quite uncommon anatomical anomaly characterized by a gallbladder that is completely or partially suspended by a mesentery, resulting in increased mobility within the abdominal cavity. This unusual configuration can predispose the organ to torsion, ischemia, or necrosis, although it often remains asymptomatic and is discovered incidentally. This report presents a rare case of a floating gallbladder identified unexpectedly during surgical intervention. We discuss the clinical presentation, diagnostic challenges, intraoperative findings, and management strategies, along with a review of potential complications associated with this condition. The rarity of this anomaly underscores the importance of awareness among clinicians, particularly surgeons, to avoid misdiagnosis and ensure prompt, appropriate management when encountered.

## Introduction

The gallbladder is typically anchored to the liver's undersurface by connective tissue and the cystic duct, which helps maintain its anatomical position and limits its mobility. A floating gallbladder, also referred to as a "wandering gallbladder", occurs when it is suspended by a mesentery, allowing it to move freely within the abdominal cavity [1,2]. This increased mobility predisposes the gallbladder to torsion, volvulus, and other complications, which can lead to ischemia and necrosis if not promptly addressed [3]. The majority of cases have been reported in the sixth to seventh decade of life with a female gender preponderance of 3:1, with the exception of a few isolated occurrences in the juvenile age range [4]. The condition is exceedingly rare, with few cases reported in the literature, making it a diagnostic challenge for clinicians. Certain articles mention the incidence as 4.4 to 4.6%; however, there is no clear data to corroborate the value [5]. Due to its rarity, there is limited awareness among healthcare professionals, which often results in delayed diagnosis and management [6]. This report aims to contribute to the existing body of knowledge by highlighting clinical features, diagnostic approaches, and surgical management of a floating gallbladder.

## Case presentation

A 39-year-old woman presented with complaints of right upper abdominal pain for two days, associated with multiple episodes of non-bilious, non-blood-stained vomiting. She had a history of fever three days prior, which subsided with medication. On physical examination, there was mild right hypochondrial tenderness, a positive Murphy’s sign, no palpable mass or organomegaly, and bowel sounds were normal. Systemic examination revealed no abnormalities.

**Figure 1:**
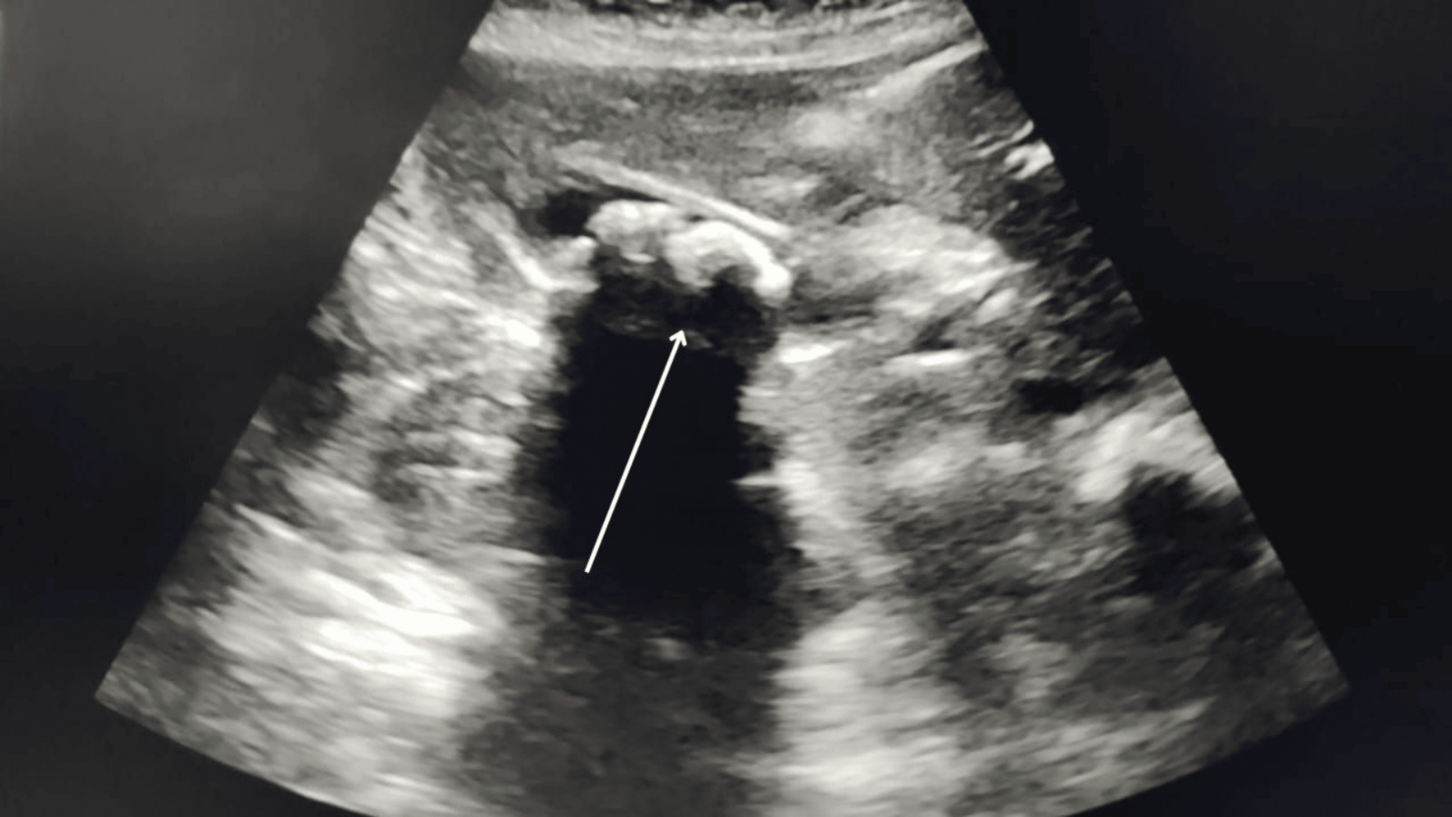
Ultrasound image showing the gallbladder with calculi (marked with a white arrow)

**Figure 2:**
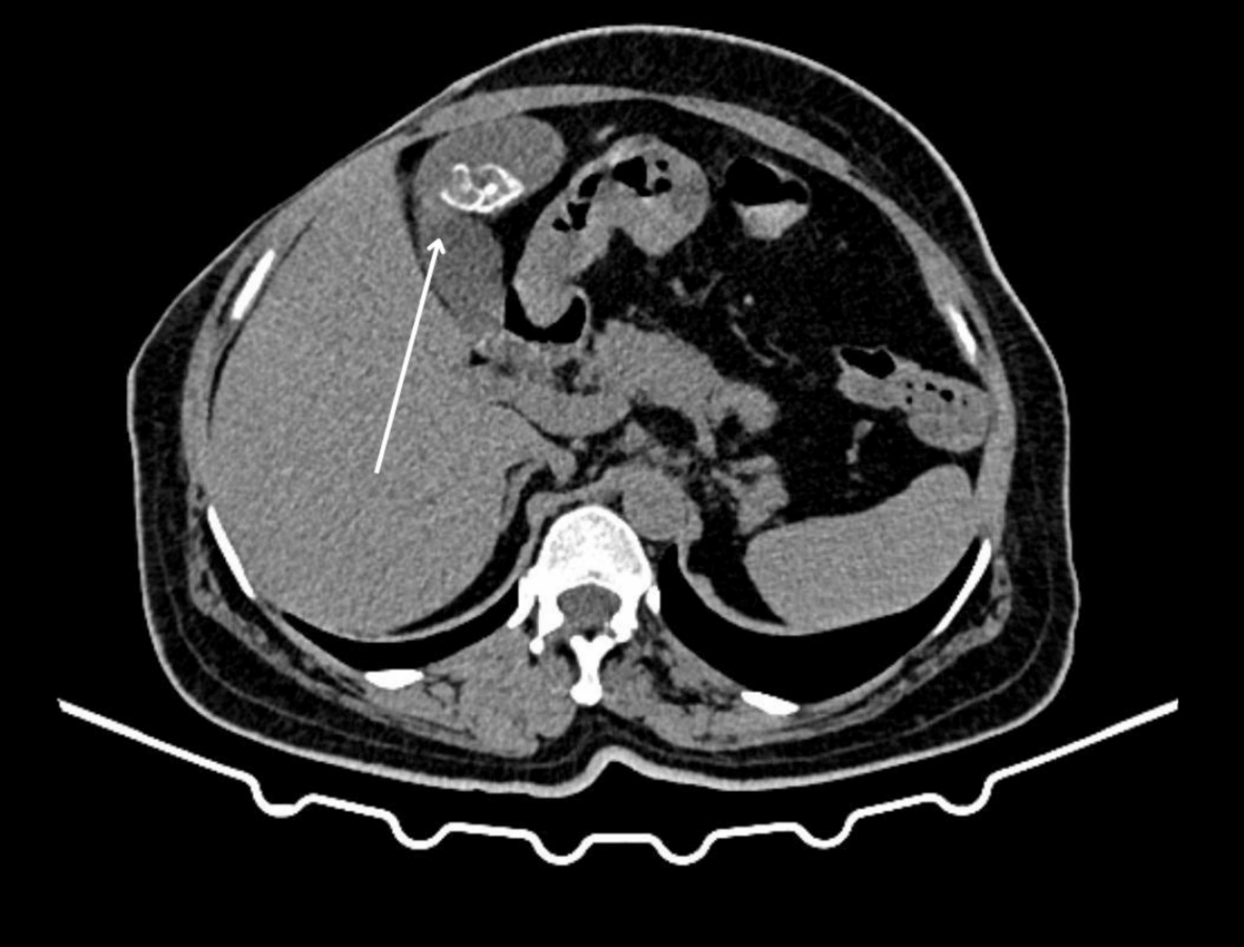
CT image showing the gallbladder with calculi (marked with a white arrow)

**Figure 3:**
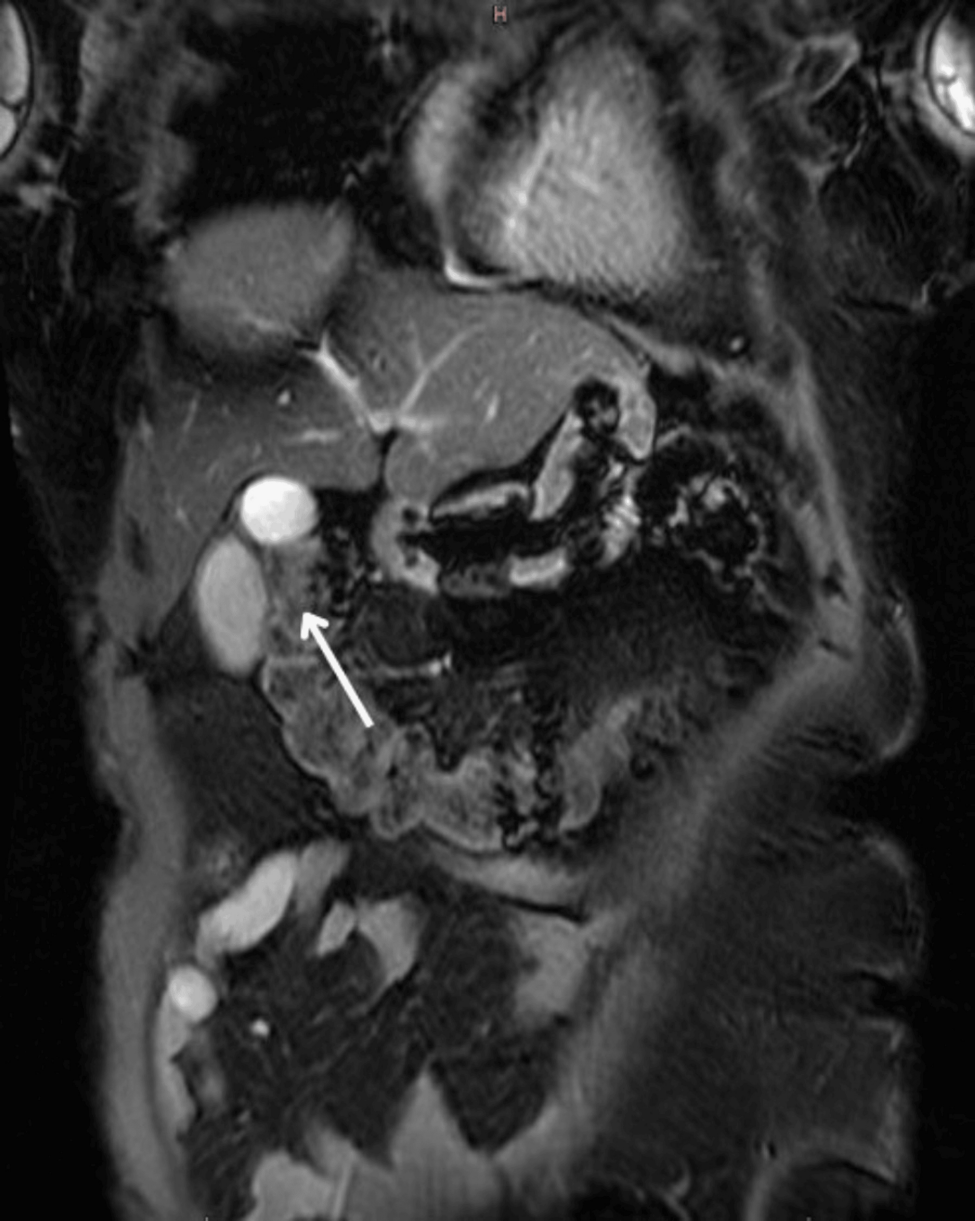
MRCP image showing the gallbladder (marked with a white arrow) MRCP: Magnetic resonance cholangiopancreatography

Ultrasound of the abdomen revealed multiple gallbladder calculi, with the largest measuring 15 mm with gallbladder thickening and pericholecystic fat-stranding (Figure 1). Contrast-enhanced CT (CECT) of the abdomen confirmed multiple calculi in the lumen and neck of the gallbladder, the largest measuring 13 × 12 mm and 4.4 × 4 mm, respectively, with a common bile duct (CBD) with a diameter of 5.7 mm (Figure 2). Magnetic resonance cholangiopancreatography (MRCP) also demonstrated multiple gallbladder calculi without evidence of CBD stones, along with an incidental finding of a duodenal diverticulum (Figure 3).

**Figure 4:**
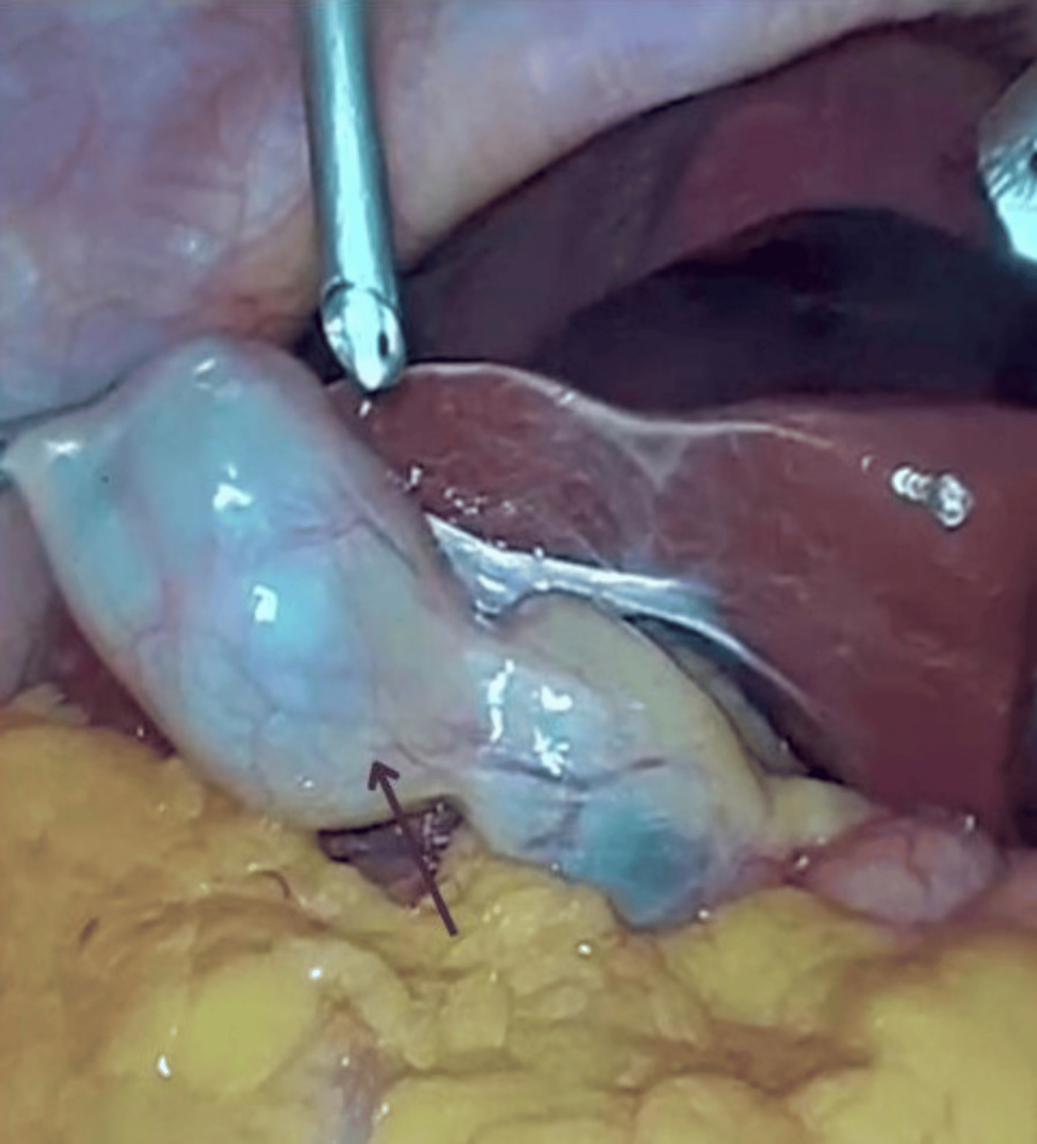
Intraoperative picture showing the floating gallbladder (marked with a black arrow)

**Figure 5:**
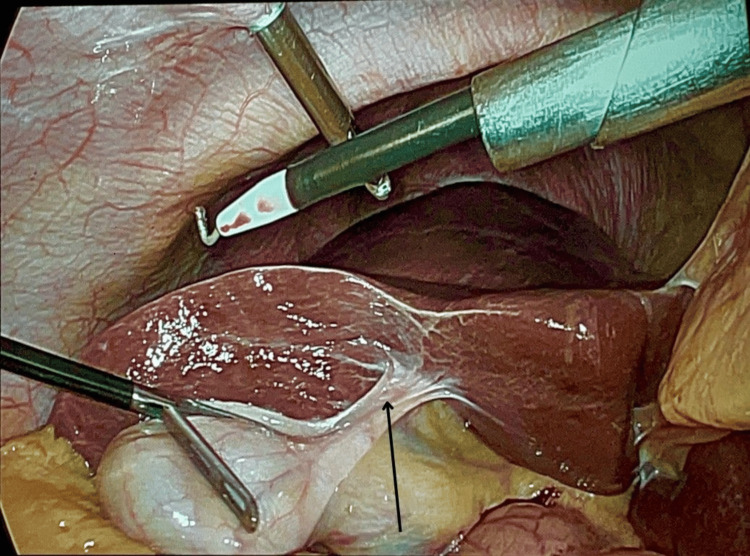
Intraoperative picture showing the floating gallbladder attached to a loose mesentery (marked with a black arrow)

The patient was provisionally diagnosed with acute calculous cholecystitis and underwent elective laparoscopic cholecystectomy. Intraoperatively, a free-floating gallbladder with twisting was noted at the junction of body and neck of gallbladder with stones in its neck. The liver anatomy appeared normal. Figures 4, 5 show the intraoperative finding of the floating gallbladder.

**Figure 6:**
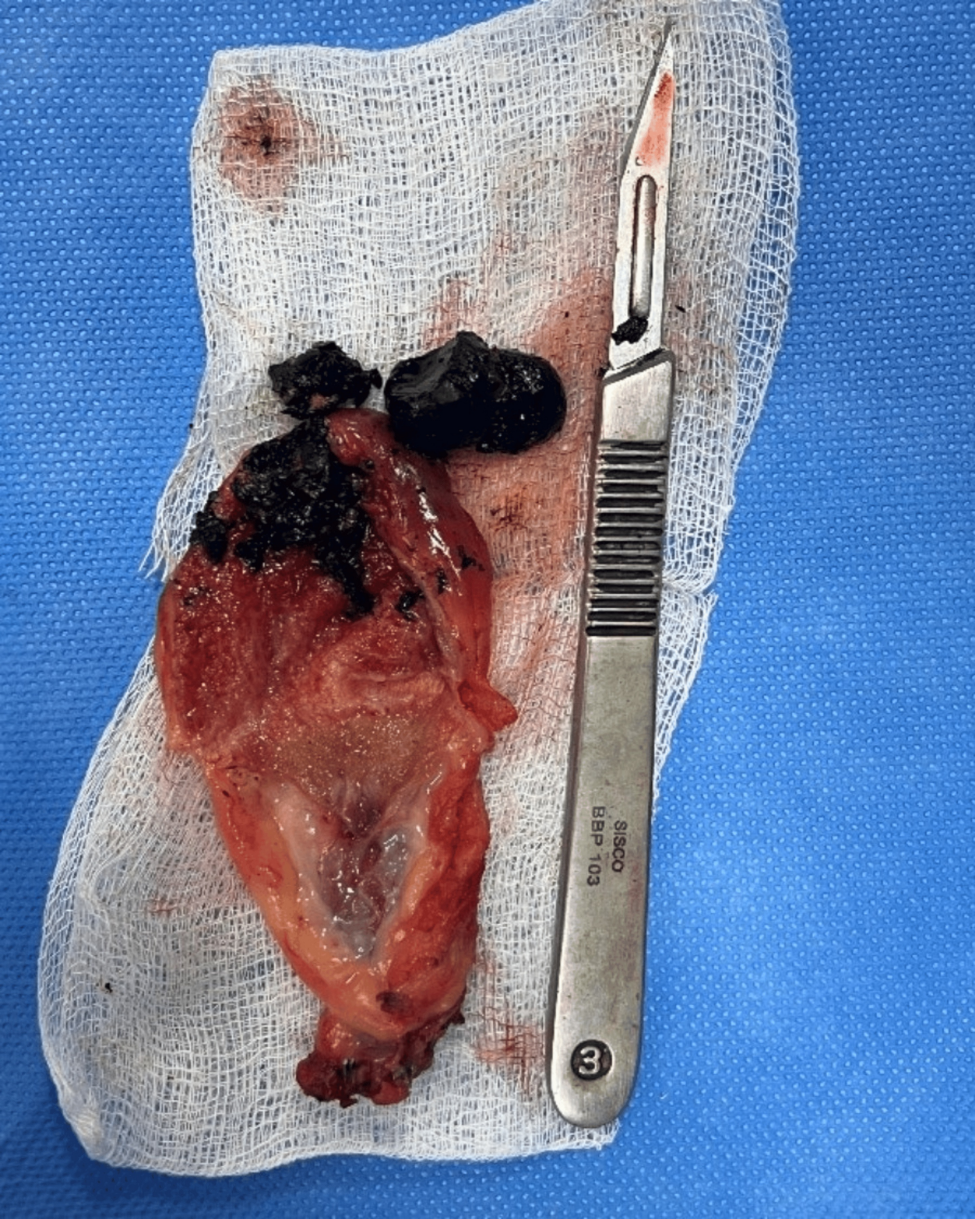
Image showing the excised gallbladder specimen

**Figure 7:**
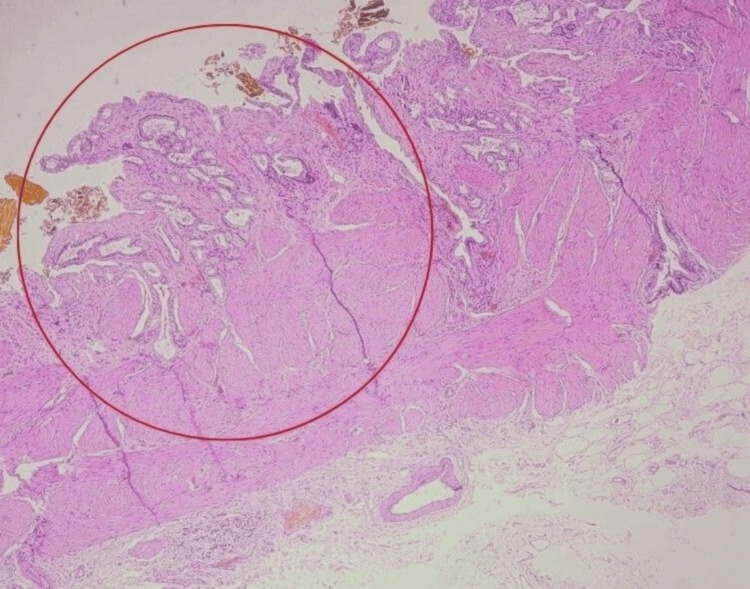
Low-power HPE slide showing features of cholecystitis (marked with a red circle) HPE: Histopathological examination

Derotation of gallbladder was done. Cholecystectomy was completed by a standard technique, and the specimen was sent for histopathological examination (HPE). Figure 6 shows the excised specimen and Figure 7 shows the HPE slide showing features of ulcerated gallbladder mucosa composed of chronic inflammatory cells infiltrates extending up to the muscularis layer consistent with chronic cholecystitis.

## Discussion

A floating gallbladder is a rare anomaly with significant clinical implications initially described by Wendel in 1898 [2]. The etiology is believed to be secondary to aberrant pars cystica migration from the hepatic diverticulum between weeks four and seven of embryologic development [7].

Its mobility increases the risk of volvulus (torsion), which can lead to gallbladder ischemia, necrosis, and gangrene if not promptly treated. The condition often mimics acute cholecystitis, presenting with symptoms such as right upper quadrant pain, nausea, vomiting, and leukocytosis, which can mislead clinicians during the initial evaluation [8].

The pathophysiology of gallbladder torsion involves the twisting of the gallbladder around its mesenteric stalk, compromising vascular supply and leading to ischemic changes [9]. Factors that may predispose to torsion include a long mesentery, sudden changes in intra-abdominal pressure, and vigorous physical activity. Although ultrasonography and CT scans can provide valuable diagnostic clues, such as an abnormally positioned gallbladder with signs of vascular compromise, definitive diagnosis is often made intraoperatively [3,9,10].

Management of a floating gallbladder primarily involves surgical intervention [11]. Laparoscopy is both diagnostic and therapeutic, offering the upper hand in minimally invasive surgery, including reduced postoperative pain and faster recovery. Early surgical intervention is critical to prevent severe complications, such as gallbladder gangrene, perforation, peritonitis, and sepsis [12]. In cases where torsion has already led to ischemia or necrosis, prompt surgery is life-saving.

## Conclusions

This case highlights the importance of considering anatomical variations like a floating gallbladder in patients presenting with atypical abdominal pain. Awareness of this rare condition can facilitate early diagnosis and appropriate surgical management, reducing the risk of severe complications. Moreover, incorporating knowledge of such rare anomalies into clinical practice can improve diagnostic accuracy, particularly in emergency settings where rapid decision-making is critical. Education with training programs should emphasize the potential for anatomical variations to influence disease presentation and outcomes. Additionally, interdisciplinary collaboration among surgeons, radiologists, and emergency physicians is vital to ensure comprehensive evaluation and prompt treatment of patients with suspected biliary anomalies. By sharing this case, we aim to contribute to a broader understanding of a floating gallbladder and encourage further research to explore its epidemiology, pathophysiology, and optimal management strategies.
